# Synthetic drug use and HIV infection among men who have sex with men in China: A sixteen-city, cross-sectional survey

**DOI:** 10.1371/journal.pone.0200816

**Published:** 2018-07-31

**Authors:** Wei Luo, Hang Hong, Xiaofang Wang, Jennifer M. McGoogan, Keming Rou, Zunyou Wu

**Affiliations:** 1 National Center for AIDS/STD Control and Prevention, Chinese Center for Disease Control and Prevention, Beijing, People’s Republic of China; 2 Department of Epidemiology, Fielding School of Public Health, University of California, Los Angeles, California, United States of America; Lewis Katz School of Medicine at Temple University, UNITED STATES

## Abstract

**Introduction:**

Increasing evidence suggests an association between synthetic drug use and HIV infection among men who have sex with men (MSM). The aim of this study was to evaluate synthetic drug use prevalence, describe characteristics of synthetic drug users, and investigate whether synthetic drug use is associated with HIV infection among Chinese MSM.

**Methods:**

A cross-sectional survey was conducted in 16 Chinese cities among males >18 years old who reported having had sex with men in the prior 3 months, but did not already have a known HIV-positive serostatus. Participants were grouped according to lifetime synthetic drug use and characteristics were compared using Chi-square test. Determinants of HIV infection were assessed using univariate and multivariate regression.

**Results:**

Among 3,135 participants, 1,249 reported lifetime synthetic drug use, for a prevalence of 39.8%. Nearly all users (96.3%) reported using inhaled alkyl nitrites (“poppers”). Synthetic drug users were more likely to be younger (<30 years, p<0.001), single (p<0.001), and more educated (p<0.001), and were more likely to engage in higher risk sexual behavior compared to non-drug users. Overall HIV prevalence was 7.8% (246/3,135). However, prevalence among synthetic drug users was 10.6% (132/246) compared to 6.0% (114/246) for non-drug users (p<0.001). Factors associated with an increased odds of HIV infection included inconsistent condom use with male partners (adjusted odds ratio [OR] = 2.18, 95% confidence interval [CI] = 1.64–2.91) and synthetic drug use (adjusted OR = 2.04, CI = 1.56–2.70).

**Conclusion:**

Prevalence of synthetic drug use, especially poppers use, prevalence was high in our study, and users had 2-fold greater odds of HIV acquisition. It is clear that there is an urgent need for increased prevention, testing, and treatment interventions for this key, dual-risk population in China. Moreover, we call on the Chinese Government to consider regulating poppers so that users can be properly warned about their associated risks.

## Introduction

Certain key populations experience higher risk of human immunodeficiency virus (HIV) infection compared to the general population across all geographies, worldwide. For men who have sex with men (MSM), risk of HIV infection is 24-fold greater [1]. Stigma, discrimination, and violence associated with HIV infection cause this population to remain “hidden,” resulting in poorer uptake of HIV prevention, testing, treatment, and care, thereby creating a major, global public health challenge [2–4].

Over the past three decades, researchers have consistently documented an array of sexual behaviors among MSM that have contributed to this key population’s high risk for HIV infection[3]. However, new evidence from Europe [5], North America [6], Australia [7], and Asia [8], suggests that drug use is further elevating the risk for HIV infection for this vulnerable population [9–11]. While drug use among MSM has historically involved the use of stimulants (e.g., ecstasy) or dissociatives (e.g., ketamine), other synthetic drugs such as methamphetamine, mephedrone, and alkyl nitrites (so-called “poppers”) are becoming more popular since users tend to experience both stimulant and sexual effects—disinhibition and euphoria combined with enhanced libido, arousal, and pleasure[9, 10].

In China, both the drug use and HIV epidemics have continued to grow at a rapid rate. The number of drug users registered with the Chinese Government doubled from 2010, to nearly 3 million in 2014. However, the actual number was estimated to be more than 14 million in 2014. The variety of drugs used is also expanding from the traditional opium and heroin to amphetamine-type stimulants (e.g., methamphetamine) and new psychoactive substances (e.g., poppers). The prevalence of synthetic drug use among registered drug users in China exceeded that of heroin and opium use for the first time in 2014[12]. As for China’s HIV epidemic, in 2015, a total of 574,000 people had been diagnosed with HIV infection, yet an additional 275,000 people were estimated to have HIV infection but still be unaware of it. The numbers of new infections are rising, with over 115,000 newly-diagnosed infections in 2015 alone [13]. Furthermore, the key population with the most rapidly growing HIV prevalence is MSM—roughly 1.5% in 2005, 6% in 2010, and 8% in 2015[13, 14]. Although the intersection of the two epidemics began with unsafe injecting behavior among heroin users in rural southwestern China [13], a new overlap has developed—high-risk sexual behavior among MSM who use synthetic drugs in China’s cities.

Several studies have sought to examine this issue [15–21], but most have been limited by their small sample sizes and/or geographical settings. Therefore, our aim was to evaluate the prevalence of synthetic drug use, to describe the characteristics of people who use synthetic drugs, and to investigate whether synthetic drug use was associated with HIV infection among a large sample of MSM across 16 cities in China. For the purposes of this study, we defined synthetic drugs as including ecstasy; ketamine; methamphetamine; Magu, which is a mix of methamphetamine and caffeine; and alkyl nitrite inhalants, also known as poppers. This group of drugs is often referred to by other terms such as “recreational drugs” or “club drugs.”

## Materials and methods

### Study design and setting

**Fig 1:**
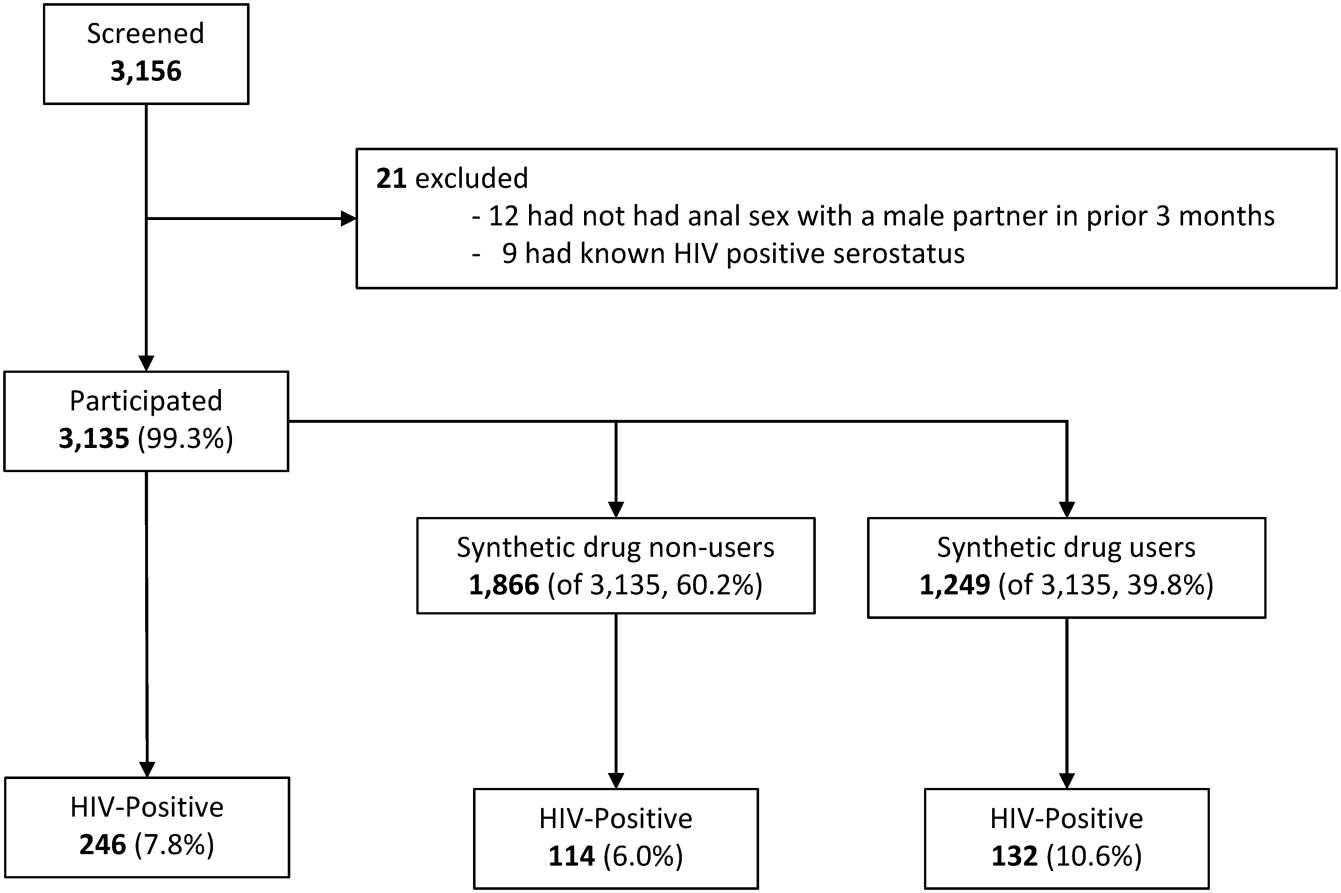
Flow diagram depicting the design of the cross-sectional study conducted among MSM in China. A 39.8% lifetime synthetic drug use prevalence was observed. Overall HIV prevalence was 7.8%, with a higher prevalence among those who had used synthetic drugs in their lifetimes (10.6%) compared to those who had never used these drugs (6.0%).

A cross-sectional study was conducted in 16 Chinese cities (Beijing, Tianjin, Changchun, Harbin, Shanghai, Nanjing, Hangzhou, Wuhan, Guangzhou, Shenzhen, Nanning, Chengdu, Chongqing, Kunming, Xi’an, and Wulumuqi) from July to September 2015. Fig 1 contains a flow diagram depicting the study design.

Study cities were selected based upon their having strong MSM community-based organizations (CBOs) that were providing HIV counseling and testing services at the time of the study. The largest MSM CBO in each selected city was chosen as that city’s study site.

### Participants

Attendees of study CBOs were screened for study participation. Eligibility criteria were: (1) being male, (2) being >18 years of age, and (3) having had anal sex with at least one male partner in the 3 months prior. Potential participants who met these criteria, but who had a known HIV-positive serostatus were excluded from the study. Individuals with diagnosed HIV infection were excluded from the study because awareness of HIV-positive serostatus may have influenced their HIV-related risk behavior, confounding our ability to investigate whether synthetic drug use was associated with HIV infection.

### Sample size

Target sample size was calculated using the formula, $n\;=\;\frac{{\left(u_{\alpha }+u_{\beta }\right)}^{2}\times 4\pi_{c}(1-\pi_{c})}{{\left(\pi_{1}-\pi_{2}\right)}^{2}}$, where *α* = 0.05, *β* = 0.1, *u*_*α*_ = 1.64, *u*_*β*_ = 1.28, and *π*_1_ and *π*_2_ were HIV prevalence rates among MSM who used drugs and MSM who did not. We assumed that *π*_1_ = 10%, *π*_2_ = 5%, and therefore $\pi_{c}\;=\;\frac{\left(\pi_{1}-\pi_{2}\right)}{2}\;=\;7.5\%$. Using this formula, we determined that a sample size of 946 would be required to detect a statistically significant difference between two groups (473 each). However, we estimated that the proportion of MSM who had used synthetic drugs in their lifetimes was approximately 25%, which elevated our sample size requirement to 1,900. We further assumed a participation refusal rate of 10%, again increasing our sample size requirement, this time to 2,100.

### Face-to-face interviews

All interviews were conducted face-to-face in a private room at CBO offices by trained CBO staff members using a structured questionnaire. The questionnaire was intended to collect information from participants on demographic, sexual behavior (in the prior 3 months), and drug use behavior (lifetime) characteristics. The term synthetic drugs was defined for participants as including ecstasy, ketamine, methamphetamine, Magu, and “poppers.” Participants were allowed to respond freely to all demographic, sexual behavior history, and drug use behavior history questions. No pre-determined answer choices were offered, and answers were not independently verified. After each interview was completed, interviewers coded responses into categories for each variable.

### Specimen collection and laboratory testing

Each participant was asked to provide a single 5 mL specimen of venous blood for HIV testing at the time of their interview. All specimens were collected by a trained nurse and stored at room temperature until centrifuged (within 12 hours of collection) to harvest serum. All serum samples were cryopreserved immediately for later HIV testing. Laboratory testing was performed at municipal HIV reference laboratories in compliance with China’s National HIV Testing Guidelines [22]. HIV screening was first conducted by ELISA (Kehua Biotech Co. Ltd, Shanghai, China) and reactive samples were re-tested by two ELISAs (Kehua Biotech Co. Ltd, Shanghai, China and Acon Biotech Co. Ltd, Hangzhou, China) run in parallel. Confirmatory testing by Western blot (Genelabs Diagnostics, Singapore) was conducted on all samples yielding at least one positive ELISA result. All participants were notified of their results and given post-test counseling by trained CBO staff. All HIV-positive participants were referred to treatment.

### Statistical analysis

Categorical variables were presented as number and percent. Prevalence of synthetic drug use was calculated by the number of participants reporting lifetime use (numerator) divided by the total number of participants (denominator). Similarly, HIV prevalence was calculated by the number of participants with HIV-positive serostatus divided by the total participants. Both were expressed as percentages. Participants were grouped according to whether they had or had not used synthetic drugs in their lifetimes. Characteristics of participants in these two subgroups (users versus non-users) were compared using Chi-square test.

Multiple logistic regression was used to investigate whether synthetic drug use was associated with HIV infection, controlling for potential confounding factors including demographic characteristics and risk behaviors. Demographic characteristics included Age (≥30 years vs <30 years as the reference), Ethnicity (Other vs Han as the reference), Marital Status (Married/cohabitating, Divorced/widowed vs Single as the reference), Education Level (≥College vs ≤High school as the reference), Monthly Income (≥CNY 3,000 vs <CNY 3,000 as the reference). Risk behaviors included Frequency of Male-Male Sexual Behaviors (≥1 time per week vs <1 time per week as the reference), Number of Male Sexual Partners (≥2 vs 1 as the reference), Participated in Male Group Sex (No vs Yes as the reference), Duration of Penetrative Sexual Behavior (≥20 minutes vs <20 minutes as the reference), Consistent Condom Use with Male Partners (No vs Yes as the reference), Synthetic Drug User (Yes vs No as the reference).

All variables were included in the univariate analysis. Variables found to have significance in the univariate analysis were included in the multiple logistic regression. In addition, variables not found to have significance in the univariate analysis, but were believed to be important were also included in the final multivariate logistic regression model. A stepwise algorithm was used to select variables to enter and remain in the model, where the SLENTRY = 0.05 (i.e., only variables being significant at the 0.05 level can be added to the model) and the SLSTAY = 0.15 (i.e., variables being significant at the 0.15 level can remain in the model).

All confidence intervals (CIs) presented were 95% CIs. All p-values were 2-sided. P-values less than 0.05 were considered statistically significant. Data were double entered and verified using EpiData software (Version 3.0, The Epidata Association, Denmark). Data analyses were performed using SAS software (Version 9.1, SAS Institute, USA).

### Ethical considerations

The study protocol was reviewed and approved by the Institutional Review Board of the National Center for AIDS/STD Control and Prevention, Chinese Center for Disease Control and Prevention. Written informed consent was obtained from all participants prior to enrollment. All data collected, including HIV test results, were kept confidential. Each participant was compensated CNY 30 (approximately USD 5).

## Results and discussion

### Participants

As shown in Fig 1, a total of 3,156 individuals seeking HIV counseling and testing services at selected CBOs in the 16 cities were screened for study participation. Among those screened, 12 failed to meet inclusion criteria and 9 were excluded due to known HIV-positive serostatus. Thus, a total of 3,135 (99.3%) enrolled in the study, and all completed the questionnaire and HIV testing.

Characteristics of study participants are shown in Table 1. A majority of participants were younger than 30 years of age (58.1%), were of a Han ethnicity (91.4%), were single (72.2%), had an education level of college or above (61.7%), and reported earning a monthly income of at least CNY 3,000 (approximately USD 430; 63.9%). In terms of sexual behavior, a majority of participants reported having engaged in male-male sexual behaviors less than once per week (61.0%), having more than one male sexual partner (56.6%), not participating in male group sex (95.0%), having penetrative sexual behavior durations of ≥20 minutes (57.5%), and inconsistently using condoms with male sexual partners (53.3%). A total of 246 participants (of 3,135) were found to have an HIV-positive serostatus, for an overall study population HIV prevalence of 7.8%.

**Table 1:** Characteristics of all participants, and participants grouped by lifetime synthetic drug use.

Variables	All Participants N (%)	Synthetic Drug^a^ User Subgroup Comparison			
		Non-User n (%)	User n (%)	χ^2^ Value	P-Value^b^
**Overall**	3,135 (100)	1,886 (100)	1,249 (100)		
**Age**					
<30 years	1,822 (58.1)	991 (52.5)	831 (66.5)	60.4	<0.001
≥30 years	1,313 (41.8)	895 (47.5)	418 (33.5)		
**Ethnicity**					
Han	2,864 (91.4)	1,730 (91.7)	1,134 (90.8)	0.8	0.36
Other	271 (8.6)	156 (8.3)	115 (9.2)		
**Marital Status**					
Single	2,264 (72.2)	1,271 (67.4)	993 (79.5)	55.3	<0.001
Married/cohabitating	731 (23.3)	513 (27.2)	218 (17.5)		
Divorced/widowed	140 (4.5)	102 (5.4)	38 (3.0)		
**Education Level**					
≤High school	1,202 (38.3)	784 (41.6)	418 (33.5)	20.9	<0.001
≥College	1,933 (61.7)	1,102 (58.4)	831 (66.5)		
**Monthly Income**					
<CNY 3,000	1,132 (36.1)	697 (37.0)	435 (34.8)	1.5	0.22
≥CNY 3,000	2,003 (63.9)	1,189 (63.0)	814 (65.2)		
**Frequency of Male-Male Sexual Behaviors**					
<1 time per week	1,912 (61.0)	1,211 (64.2)	701 (56.1)	19.9	<0.001
≥1 time per week	1,220 (38.9)	675 (35.8)	545 (43.7)		
Missing	3 (0.1)	0 (0.0)	3 (0.2)		
**Number of Male Sexual Partners**					
1	1,358 (43.3)	918 (48.7)	440 (35.2)	54.5	<0.001
≥2	1,774 (56.6)	968 (51.3)	806 (64.5)		
Missing	3 (0.1)	0 (0.0)	3 (0.2)		
**Participated in Male Group Sex**					
Yes	153 (4.9)	61 (3.2)	92 (7.4)	27.8	<0.001
No	2,979 (95.0)	1,825 (96.8)	1,154 (92.4)		
Missing	3 (0.1)	0 (0.0)	3 (0.2)		
**Duration of Penetrative Sexual Behavior**					
<20 minutes	1,321 (42.1)	894 (47.4)	427 (34.2)	56.1	<0.001
≥20 minutes	1,802 (57.5)	980 (52.0)	822 (65.8)		
Missing	12 (0.4)	12 (0.6)	0 (0.0)		
**Consistent Condom Use with Male Partners**					
Yes	1,460 (46.6)	906 (48.0)	554 (44.3)	4.2	0.04
No	1,671 (53.3)	977 (51.8)	694 (55.6)		
Missing	4 (0.1)	3 (0.2)	1 (0.1)		
**HIV Serostatus**					
Positive	246 (7.8)	114 (6.0)	132 (10.6)	21.3	<0.001
Negative	2,889 (92.2)	1,772 (94.0)	1,117 (89.4)		

^a^The term “synthetic drug” included ecstasy, ketamine, methamphetamine, Magu (combination of methamphetamine and caffeine), and “poppers” (inhaled alkyl nitrites).

^b^Subgroups were compared using χ^2^ test to generate p-values listed.

### Synthetic drug users versus non-users

While 1,886 participants (of 3,135, 60.2%) reported never having used synthetic drugs in their lifetimes, a total of 1,249 had used synthetic drugs at least once before, for an overall lifetime synthetic drug use prevalence of 39.8% (Fig 1). Among those who reported having used synthetic drugs in their lifetimes, 96.3% (1,203/1,249) reported having used poppers and 3.7% (46/1,203) reported having used other synthetic drugs such as methamphetamine, ketamine, ecstasy, and Magu (data not shown).

A comparison of these two groups is presented in Table 1. A greater proportion of synthetic drug users were young (<30 years, χ^2^ = 60.4, p<0.001), single (χ^2^ = 55.3, p<0.001), and had at least some college education (χ^2^ = 20.9, p<0.001), compared to non-users. A greater proportion of synthetic drug users reported having engaged in male-male sexual behavior more than once per week (χ^2^ = 19.9, p<0.001), having had more than one male sexual partner (χ^2^ = 54.5, p<0.001), having participated in male group sex (χ^2^ = 27.8, p<0.001), and having had penetrative sexual behavior durations of ≥20 minutes (χ^2^ = 56.1, p<0.001), and having consistently used condoms with male partners (χ^2^ = 4.2, p = 0.04), compared to non-users. Finally, compared to non-users, a greater proportion of synthetic users had an HIV-positive serostatus (χ^2^ = 21.3, p<0.001).

### Factors associated with HIV-positive serostatus

Table 2 presents the characteristics of the 246 participants diagnosed with HIV infection as well as factors associated with HIV-positive serostatus. Among these 246 participants, a majority was <30 years old (60.1%), of Han ethnicity (93.0%), single (68.7%), had education levels of college or above (50.8%), and reported monthly incomes of at least CNY 3,000 (approximately USD 430; 64.2%). Most reported having engaged in male-male sexual behaviors less than once per week (69.1%), having had more than one male sexual partner (58·1%), not having participated in male group sex (93.9%), having had penetrative sexual behavior durations of ≥20 minutes (63.0%), and having inconsistently used condoms with male sexual partners (69.5%). A majority of participants diagnosed with HIV infection had also used synthetic drugs in their lifetimes (53.7%).

**Table 2:** Results of univariate and multivariate regression models predicting factors associated with HIV-positive serostatus.

Variables	HIV-Positive Serostatus N (%)	Unadjusted OR (CI)	P-value^a^	Adjusted OR (CI)	P-value^a^
**Overall**	246 (100)				
**Age**					
<30 years	148 (60.1)	1.00		1.00	
≥30 years	98 (39.8)	0.91 (0.70–1.19)	0.50	0.77 (0.56–1.07)	0.12
**Ethnicity**					
Han	229 (93.0)	1.00		1.00	
Other	17 (6.9)	0.77 (0.46–1.28)	0.32	0.72 (0.43–1.23)	0.23
**Marital Status**					
Single	169 (68.7)	1.00		1.00	
Married/cohabitating	68 (27.6)	1.27 (0.95–1.71)	0.11	1.37 (0.97–1.95)	0.08
Divorced/widowed	9 (3.7)	0.85 (0.43–1.70)	0.65	0.86 (0.41–1.82)	0.70
**Education Level**					
≤High school	121 (49.2)	1.00		1.00	
≥College	125 (50.8)	0.62 (0.48–0.80)	<0.001	0.54 (0.41–0.71)	<0.001
**Monthly Income**					
<CNY 3,000	88 (35.8)	1.00		1.00	
≥CNY 3,000	158 (64.2)	1.02 (0.77–1.33)	0.91	1.08 (0.81–1.43)	0.61
**Frequency of Male-Male Sexual Behaviors**					
<1 time per week	170 (69.1)	1.00		1.00	
≥1 time per week	76 (30.9)	0.68 (0.51–0.90)	0.007	0.55 (0.41–0.73)	<0.001
**Number of Male Sexual Partners**					
1	103 (41.9)	1.00		1.00	
≥2	143 (58.1)	1.07 (0.82–1.39)	0.62	0.93 (0.70–1.24)	0.63
**Participated in Male Group Sex**					
Yes	15 (6.1)	1.00		1.00	
No	231 (93.9)	0.78 (0.45–1.33)	0.36	0.90 (0.50–1.60)	0.72
**Duration of Penetrative Sexual Behavior** behavior					
<20 minutes	89 (36.2)	1.00		1.00	
≥20 minutes	155 (63.0)	1.30 (0.99–1.71)	0.06	1.21 (0.92–1.60)	0.17
Missing	2 (0.8)				
**Consistent Condom Use with Male Partners** sex with male					
Yes	75 (30.5)	1.00		1.00	
No	171 (69.5)	2.10 (1.59–2.78)	<0.001	2.18 (1.64–2.91)	<0.001
**Synthetic Drug**^b^ **User**					
No	114 (46.3)	1.00		1.00	
Yes	132 (53.7)	1.85 (1.41–2.38)	<0.001	2.04 (1.56–2.70)	<0.001

OR: odds ratio; CI: 95% confidence interval.

^a^ORs, CIs, and p-values generated based on univariate and multivariate logistic regression model.

^b^The term “synthetic drug” included ecstasy, ketamine, methamphetamine, Magu (combination of methamphetamine and caffeine), and “poppers” (inhaled alkyl nitrites).

After controlling for possible confounding factors, multivariate regression analysis revealed two factors associated with higher odds of HIV-positive serostatus: inconsistent condom use with male partners (adjusted OR = 2.18, CI = 1.64–2.91) and lifetime synthetic drug use (adjusted OR = 2.04, CI = 1.56–2.70). Two factors were found to be protective: higher educational attainment (≥college: adjusted OR = 0.54, CI = 0.41–0.71) and higher frequency of male-male sexual behaviors (≥1 time per week: adjusted OR = 0.55, CI = 0.41–0.73, Table 2).

### Interpretations

Our main findings were an overall lifetime synthetic drug use prevalence of 39.8% among the 3,156 MSM surveyed across 16 cities in China. Synthetic drug users were more likely to be young, single, and better educated, and were more likely to engage in higher risk sexual behavior compared to non-users. HIV prevalence among synthetic drug users was 10.6%, well above that of non-users (6.0%), and odds of HIV-positive serostatus was 2-fold higher among synthetic drug users, compared to non-users.

Our finding of nearly 40% prevalence of lifetime synthetic drug use was higher than the 23% observed in a 2011 study in Shenyang,[20] and the 28% observed in a 2013 study in seven cities (Shanghai, Nanjing, Changsha, Zhengzhou, Ji’nan, Shenyang, and Kunming) [21]. However, our study, using more recent data from a larger sample and a broader geography, may have produced both a more representative and generalizable estimate. Furthermore, other studies published to date have examined only recent synthetic drug use (in the prior 3, 6, or 12 months) [15, 17–19], which likely underestimated of the size of the synthetic drug-using MSM population in China.

We found that inhaled alkyl nitrites, or poppers, was by far the most commonly used synthetic drug among the participants in our study (96% of users reported popper use). This puts prevalence of lifetime poppers use in our study at 38%, similar to the 47% lifetime popper use prevalence among MSM in Beijing observed in a 2012 study [16]. Poppers have previously been identified as the most popular synthetic drug among Chinese MSM in several studies [15, 17–20]. This is likely due, at least in part, to their still-legal status, ubiquitous availability, and low cost. However, it has also been attributed to the relaxation effect poppers have on rectal muscle, which reduces pain during sex. Thus, poppers provide the stimulant and sexual effects of other synthetic drugs (i.e., euphoria, disinhibition, enhanced libido, arousal, pleasure), and more, all for less money, added convenience, and no legal risk [17, 19, 20]. Finally, since poppers are legal, heavily marketed with colorful packaging, and are not labeled like drugs, carrying no visible warnings about their risks, users do not consider them dangerous.

Several studies of synthetic drug use among MSM in China have, similar to our findings, reported that MSM users of synthetic drugs tend to be young and single [15–17, 19, 20]. and have higher levels of educational attainment [16, 17]. Furthermore, several studies support our finding of increased high-risk sexual behavior among synthetic drug users compared to non-users—users being more likely to have increased numbers of male sex partners, engage in commercial sex and group sex, and have unprotected sex [15, 17, 19, 20].

Similarly, our finding of higher HIV prevalence among synthetic drug users (10.6% compared to 6.0% among non-users) and 2-fold greater odds of HIV infection among synthetic drug users is also supported by the existing literature. A 2009 study among MSM in Changsha (n = 826) found HIV prevalence of 19% among users versus 11% among non-users and 2-fold greater odds of HIV infection among users [19]. In a 2011 study in Shenyang (n = 625) HIV prevalence was higher among users (20% versus 7%) and users had 3.5-fold greater odds of HIV infection [20]. In a 2012 study in Beijing (n = 400) synthetic drug use was associated with 3-fold greater odds of HIV infection [16]. A seven-city study completed in 2013 (n = 3,830) found higher HIV prevalence among users compared to non-users (12% versus 8%) [15]. In its larger companion study (n = 4,496) conducted in the same seven cities by the same research team also in 2013, 2-fold higher odds of recent HIV infection was found among MSM who had used synthetic drugs in the prior 6 months [21]. Finally, in 2014, another study in Beijing (n = 3,588) also found synthetic drug use to be associated with greater odds of HIV infection [17].

Taken together, this evidence clearly describes synthetic drug-using MSM as a dual-risk key population in China that is very vulnerable to HIV infection. While relatively new to China, the combination of synthetic drug use and high-risk sexual behavior by MSM is not new globally [11], and in fact, has become so common that it has been given names: “party-and-play” in North America and Australia, and “chemsex” in the remainder of the West as well as parts of southern Asia and central Europe [9, 10]. Well-documented on four continents [5–8], synthetic drug use and high-risk sexual behavior has been consistently linked with HIV infection in MSM communities and is a serious global public health problem.

Between the time our study was conducted and our manuscript was submitted for publication, several additional, similar studies have been published. A large cross-sectional survey conducted in 2013 in Beijing found prevalence of recent (last 3 months) substance use to be 28%, nearly all reporting popper use, and substance users had higher odds of sexual risk behaviors including condomless anal intercourse (receptive and insertive), greater than 10 male sex partners lifetime, and multiple male partners in the past 3 months. Substance use was also associated with greater odds of HIV infection [23]. A nationwide, online, cross-sectional survey in 2014 found a 77% prevalence of lifetime synthetic drug use, and a 21% prevalence of popper use in the prior 12 months. Poppers users were more likely to have engaged in group sex in the prior 3 months and more likely to have had an HIV test [18]. A cross-sectional study in Nanjing (2014–2015) found lifetime popper use prevalence of as high as 28% and 1.7-fold greater odds of HIV infection among popper users [24]. A small survey conducted in 2016 among MSM in Tianjin found prevalence of recent (last 6 months) substance use to be approximately 100%, with recent popper use at 98%. Among popper users, more than 85% reported increased sexual desire and pleasure after using and nearly 60% reported prolonged duration of sexual activity after using [25].

So, how do we meet the challenge of HIV prevention among the synthetic drug-using MSM population? Modeling work suggests that substantial modification of high-risk sexual behavior among MSM is insufficient to drive a meaningful reduction in HIV transmission [3]. This is supported by a recent meta-analysis of studies among MSM in China, which found that although education and behavioral interventions had improved consistent condom use, HIV/AIDS knowledge, and testing uptake, it had not been enough to reduce HIV prevalence [26]. Instead, due to already-high HIV prevalence in MSM communities, high transmission probability, and force of infection, biological interventions that successfully reduce infectiousness are far more likely to curb the growth of the HIV epidemic in this population [3]. Thus, HIV pre-exposure prophylaxis (PrEP), frequent testing (including self-testing), as well as immediate ART for infected individuals with counseling and adherence support should be implemented along with increased or improved programs targeting education and behavior. However, careful implementation of these interventions will be critical since the details of how these interventions are implemented are necessarily highly contextual [27].

For MSM in China, these programs must be customized to fit within their unique lifestyles (i.e., be easy and convenient), to meet their needs for prevention of stigma and discrimination (i.e., ensure privacy and confidentiality), and to reach them at the gateway to this part of their lives (i.e., via the internet and social media). Development and delivery of a comprehensive package of PrEP, high-frequency testing including self-testing, immediate treatment, and on-going counseling and adherence support interventions that is easy, convenient, private, confidential, and demand-driven via internet and social media environments frequented by the MSM community is urgently needed. Additionally, more must be done to educate MSM on the dangers of synthetic drug use, especially popper use, and China should strongly consider regulating poppers such that they clearly display warnings regarding their dangerous effects.

### Limitations

Our study had some limitations. First, causal relationships cannot be assessed because of the cross-sectional study design. Second, the study only recruited among those who attended study CBOs to receive HIV counseling and testing services thus subjecting it to selection bias. Third, since poppers are legal in China, it is possible that their use relative to other synthetic drugs was overestimated due to social desirability and miss-classification bias. Fourth, there may have been other, unmeasured factors (e.g., alcohol and/or tobacco use) that contributed to the association between HIV infection and synthetic drug use in our study population. Lastly, there remains substantial stigma in China toward MSM and toward drug users and therefore, other participant responses may have also been subject to social desirability bias. However, considerable effort was made to prevent this by involving MSM CBO staff members in recruitment and interviewing, conducting interviews privately, assuring participants of data confidentiality, and ensuring that the questionnaire was well designed and interviewers well trained.

## Conclusions

In conclusion, our findings of synthetic drug use prevalence of nearly 40% among urban Chinese MSM, and a 2-fold greater probability of HIV-positive serostatus for this group, provide strong evidence of the pressing need of a carefully-designed and well-implemented package of biological prevention measures for this key, dual-risk population in China. Furthermore, there is now a wealth of observational evidence supporting these findings. Hence, prospective study intended to gain an understanding causality is now very much needed. Prevalence of HIV among urban Chinese MSM is already known to be on the rise [13], some reports putting prevalence figures at nearly 20% in some cities [28]. China must swiftly and effectively work to deflect the trajectory of its HIV epidemic among MSM, especially among those who use synthetic drugs, if it is to meet the UNAIDS 90-90-90 goals [29], and to eventually bring its HIV epidemic under control.

## Supporting information

S1 Data All data. (RAR)
